# Geographic Distribution of Raccoon Roundworm, *Baylisascaris procyonis*, Germany and Luxembourg

**DOI:** 10.3201/eid2604.191670

**Published:** 2020-04

**Authors:** Mike Heddergott, Peter Steinbach, Sabine Schwarz, Helena E. Anheyer-Behmenburg, Astrid Sutor, Annette Schliephake, Diana Jeschke, Michael Striese, Franz Müller, Elisabeth Meyer-Kayser, Michael Stubbe, Natalia Osten-Sacken, Susann Krüger, Wolfgang Gaede, Martin Runge, Lothar Hoffmann, Hermann Ansorge, Franz J. Conraths, Alain C. Frantz

**Affiliations:** Musée National d'Histoire Naturelle, Luxembourg, Luxembourg (M. Heddergott, P. Steinbach, A.C. Frantz);; Georg-August University, Göttingen, Germany (P. Steinbach);; Friedrich-Loeffler-Institut, Greifswald-Insel Riems, Germany (S. Schwarz, A. Sutor, F.J. Conraths);; Lower Saxony State Office for Consumer Protection and Food Safety, Hannover, Germany (H.E. Anheyer-Behmenburg, M. Runge);; State Office for Consumer Protection Saxony-Anhalt, Stendal, Germany (A. Schliephake, W. Gaede); Senckenberg Museum of Natural History Görlitz, Görlitz, Germany (D. Jeschke, M. Striese, H. Ansorge);; Justus-Liebig-University Giessen, Giessen, Germany (F. Müller);; Thuringia Office for Consumer Protection, Bad Langensalza, Germany (E. Meyer-Kayser, L. Hoffmann);; Martin-Luther University Halle-Wittenberg, Halle/Saale, Germany (M. Stubbe);; Nicolaus Copernicus University, Toruń, Poland (N. Osten-Sacken);; Fondation Faune-Flore, Luxembourg (N. Osten-Sacken);; German Hunting Association, Berlin, Germany (A. Sutor, S. Krüger);; International Institute Zittau, Technische Universität Dresden, Zittau, Germany (H. Ansorge)

**Keywords:** Ascaridida infestation, Baylisascaris procyonis, introduced species, Luxembourg, nematodes, raccoons, Germany, parasites, parasitic diseases, zoonoses

## Abstract

Infestation with *Baylisascaris procyonis*, a gastrointestinal nematode of the raccoon, can cause fatal disease in humans. We found that the parasite is widespread in central Germany and can pose a public health risk. The spread of *B. procyonis* roundworms into nematode-free raccoon populations needs to be monitored.

The raccoon roundworm (*Baylisascaris procyonis*) is a gastrointestinal parasitic nematode of the raccoon (*Procyon lotor*). It is common in its native range in North America, where its prevalence in raccoons can reach 82% (*1*). Through their feces, infested raccoons can shed millions of *B. procyonis* eggs, which may remain infective in the environment for years (*2*). Paratenic hosts can acquire the parasite when ingesting nematode eggs from raccoon latrines (*3*).

*B. procyonis* infestations are usually benign in the raccoon but can be fatal in paratenic hosts, including humans (*1*). Since 1980, several fatal cases of neural larva migrans have occurred in humans in the United States (*3*); infants have been frequently affected because of fecal–oral transmission (*4*). Increasing raccoon densities in close proximity to humans has increased public health concern about *B. procyonis* roundworms (*2*).

As a result of joint translocation with raccoons, *B. procyonis* roundworms have increased their geographic range (*5*). Raccoons are common in Germany and Luxembourg (Figure, panel A). All raccoons in Germany are assumed to have stemmed from a small number of founders and 2 separate introduction events in western Germany (Hesse) during the 1930s and eastern Germany (Brandenburg) during the 1940s (*8*). However, genetic analysis has inferred a minimum of 5 founder events (*6*). In addition to 2 genetic populations clustered around the known introduction sites (referred to as the Hesse and Brandenburg populations), distinct raccoon populations were identified in Saxony (eastern Germany), around the Harz Mountains in central Germany, and in Luxembourg and neighboring regions (Figure, panel B).

*B. procyonis* roundworms occur in the Hesse and Harz populations (*5*) but are absent from Brandenburg (*9*). No information is available about the remaining 2 populations in Luxembourg and Saxony. Although only a few human cases of baylisascariasis have been reported from Germany (*9*), a detailed overview of the parasite’s geographic distribution is needed to identify potential risk areas.

During 2008–2018, we collected 8,184 legally harvested or road-killed raccoons from Germany and Luxembourg (Figure, panel A), focusing on different regions every year or every few years, and investigated their intestines for the presence of *B. procyonis* roundworms. We plotted the presence of the parasite onto the 10 × 10–km ETRS89-LAEA5210 EEA reference grid, a base map provided by the European Environment Agency (https://www.eea.europa.eu/data-and-maps/data/eea-reference-grids-2). We calculated the proportion of infested raccoons for 69 of Germany’s 294 administrative districts where *B. procyonis* roundworms were present and >25 raccoons had been sampled. We generated maps by using ArcMap v.10.3 (ESRI Inc. https://www.esri.com).

**Figure:**
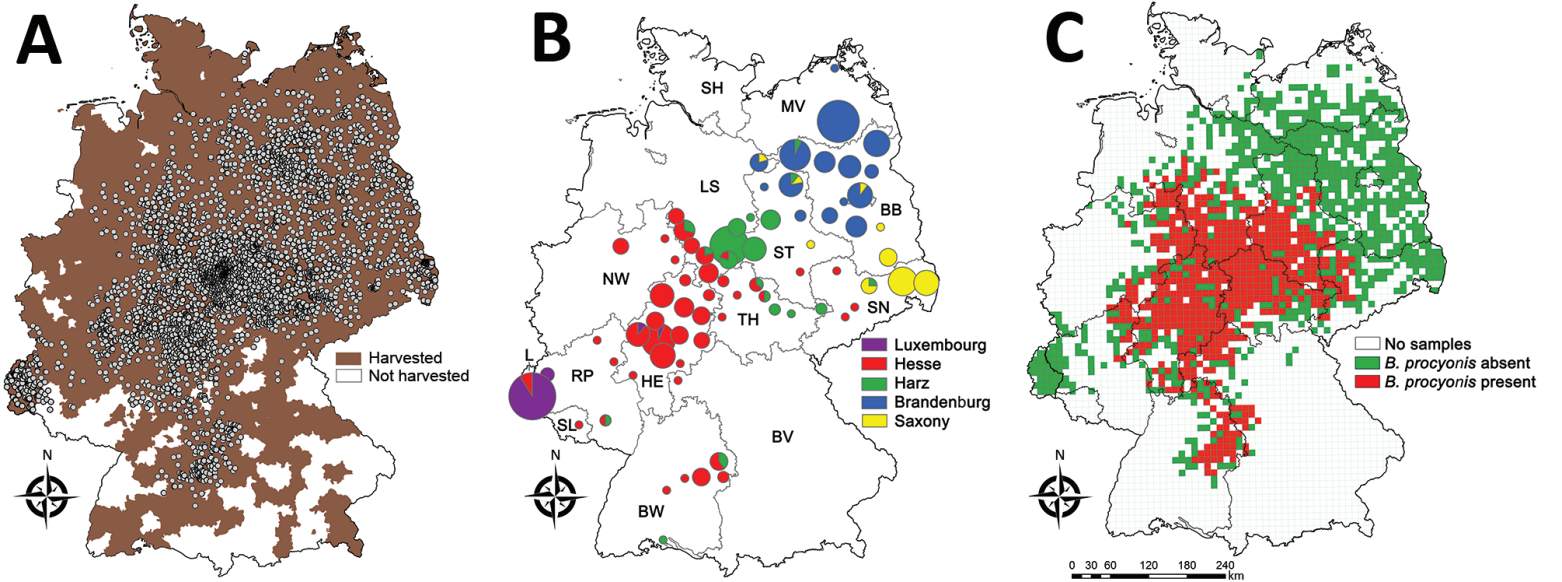
Characteristics of the geographic distribution of the raccoon roundworm (*Baylisascaris procyonis*). A) Geographic origin of 8,184 dissected raccoons and the German administrative districts (Landkreise) in which raccoons were harvested during 2017–2018. Dots indicate sampling sites. B) Population genetic structure of raccoons in Germany and Luxembourg. Reanalysis of the dataset by (*5*) but including 26 raccoons from Luxembourg (genotyped following [*5*]) and omitting animals from the city of Kassel (no distinct introduction [*6*]). The genetic data were analyzed by using the clustering of individuals algorithm implemented in BAPS v.6.0 (*7*). Different colors represent different genetic populations. Pie charts represent the genetic populations of origin of all the raccoons in an administrative district, and chart size indicates the number of samples included. BB, Brandenburg; BV, Bavaria; BW, Baden-Württemberg; HE, Hesse; L, Luxembourg; LS, Lower Saxony; MV, Mecklenburg-Western Pomerania; NW, North Rhine-Westphalia; RP, Rhineland-Palatinate; SH, Schleswig-Holstein; SL, Saarland; SN, Saxony; ST, Saxony-Anhalt; TH, Thuringia; C) Geographic distribution of *B. procyonis* roundworms, plotted onto the10 × 10–km ETRS89-LAEA5210 EEA reference grid.

*B. procyonis* roundworms were widespread in central Germany, their distribution probably corresponding to the geographic extent of the Hesse and Harz genetic populations (Figure, panels B, C). However, we did not detect the parasite in Luxembourg and western areas of Germany or in a northern/eastern region that included the federal states of Brandenburg, Mecklenburg-Western Pomerania, Schleswig-Holstein, northern parts of Lower Saxony, Saxony-Anhalt, and eastern Saxony. In other words, the parasite was not detected in the areas covered by the Luxembourg, Brandenburg, and Saxony genetic populations (Figure, panels B, C). A median of 43.6% (interquartile range 34.4%–49.7%) of raccoons were infested in the 69 administrative districts where the parasite was present and >25 raccoons had been sampled.

Identification of risk areas for human *B. procyonis* roundworm infestation is necessary because of the frequent proximity of raccoons to human populations. Our results show that the nematode is widespread and prevalent in central Germany. Given that *B. procyonis* eggs remain infective for years, the nematode is likely to pose a public health risk in its distribution area (*10*). To reduce the risk for *B. procyonis* infestation, protective measures (procedure masks, gloves, handwashing) should always be applied when raccoons or their feces in the risk area are handled. In this context, educational material should be made available to schools and daycare centers and to persons who have occupational contact with raccoons.

The match between the distribution of the roundworm and the extent of the different genetic populations of the raccoon suggests that the absence of the parasite results from the founder animals’ parasite-free status. However, we cannot exclude the possibility that ecologic or geographic differences between the introduction sites also contributed to the lack of parasites in some populations. Further research and monitoring are needed, especially in view of a possible spread of the parasite into nematode-free raccoon populations. Also, because of the rapid spread of raccoons, assessment of the status of the parasite in northwestern and southwestern Germany and at the periphery of its current distribution more generally should be considered.

## References

[1] Kazacos KR. *Baylisascaris procyonis* and related species. In: Samuels WM, Pybus MJ, Kocans AA, editors. Parasitic diseases of wild mammals. 2nd ed. Ames (IA): Iowa State University Press; 2001. p. 301–41.

[2] Page K, Beasley JC, Olson ZH, Smyser TJ, Downey M, Kellner KF, et al. Reducing *Baylisascaris procyonis* roundworm larvae in raccoon latrines. Emerg Infect Dis. 2011;17:90–3. 10.3201/eid1701.100876 21192862 PMC3204634

[3] Sorvillo F, Ash LR, Berlin OGW, Morse SA. *Baylisascaris procyonis*: an emerging helminthic zoonosis. Emerg Infect Dis. 2002;8:355–9. 10.3201/eid0804.010273 11971766 PMC2730233

[4] Pai PJ, Blackburn BG, Kazacos KR, Warrier RP, Bégué RE. Full recovery from *Baylisascaris procyonis* eosinophilic meningitis. Emerg Infect Dis. 2007;13:928–30. 10.3201/eid1306.061541 17553240

[5] Osten-Sacken N, Heddergott M, Schleimer A, Anheyer-Behmenburg HE, Runge M, Horsburgh GJ, et al. Similar yet different: co-analysis of the genetic diversity and structure of an invasive nematode parasite and its invasive mammalian host. Int J Parasitol. 2018;48:233–43. 10.1016/j.ijpara.2017.08.013 29102623

[6] Fischer ML, Hochkirch A, Heddergott M, Schulze C, Anheyer-Behmenburg HE, Lang J, et al. Historical invasion records can be misleading: genetic evidence for multiple introductions of invasive raccoons (*Procyon lotor*) in Germany. PLoS One. 2015;10:e0125441. 10.1371/journal.pone.0125441 25946257 PMC4422738

[7] Cheng L, Connor TR, Aanensen DM, Spratt BG, Corander J. Bayesian semi-supervised classification of bacterial samples using MLST databases. BMC Bioinformatics. 2011;12:302. 10.1186/1471-2105-12-302 21791094 PMC3155509

[8] Frantz AC, Cyriacks P, Schley L. Spatial behaviour of a female raccoon (*Procyon lotor*) at the edge of the species’ European distribution range. Eur J Wildl Res. 2005;51:126–30. 10.1007/s10344-005-0091-2

[9] Schwarz S, Sutor A, Mattis R, Conraths FJ. [The raccoon roundworm (*Baylisascaris procyonis*)—no zoonotic risk for Brandenburg?] [in German]. Berl Munch Tierarztl Wochenschr. 2015;128:34–8. 25876283

[10] Wise ME, Sorvillo FJ, Shafir SC, Ash LR, Berlin OG. Severe and fatal central nervous system disease in humans caused by *Baylisascaris procyonis*, the common roundworm of raccoons: a review of current literature. Microbes Infect. 2005;7:317–23. 10.1016/j.micinf.2004.12.005 15715975

